# Environmentally benign silver bio-nanomaterials as potent antioxidant, antibacterial, and antidiabetic agents: Green synthesis using *Salacia oblonga* root extract

**DOI:** 10.3389/fchem.2023.1114109

**Published:** 2023-02-03

**Authors:** Guru Kumar Dugganaboyana, Chethan Kumar Mukunda, Anisha Jain, Raghavendra Mandya Kantharaju, Rani R. Nithya, Divya Ninganna, Rathi Muthaiyan Ahalliya, Ali A. Shati, Mohammad Y. Alfaifi, Serag Eldin I. Elbehairi, Ekaterina Silina, Victor Stupin, Gopalakrishnan Velliyur Kanniappan, Raghu Ram Achar, Chandan Shivamallu, Shiva Prasad Kollur

**Affiliations:** ^1^ Division of Biochemistry, School of Life Sciences, JSS Academy of Higher Education and Research, Mysore, Karnataka, India; ^2^ Department of Biochemistry, JSS College of Arts, Commerce and Science, Mysore, Karnataka, India; ^3^ Department of Microbiology, JSS Academy of Higher Education and Research, Mysore, Karnataka, India; ^4^ Department of Biochemistry, Karpagam Academy of Higher Education, Coimbatore, Tamil Nadu, India; ^5^ Biology Department, Faculty of Sciences, King Khalid University, Abha, Saudi Arabia; ^6^ Cell Culture Lab, Egyptian Organization for Biological Products and Vaccines (VACSERA Holding Company), Giza, Egypt; ^7^ Institute of Biodesign and Modeling of Complex Systems, I.M. Sechenov First Moscow State Medical University (Sechenov University), Moscow, Russia; ^8^ Department of Hospital Surgery, N. I. Pirogov Russian National Research Medical University (RNRMU), Moscow, Russia; ^9^ School of Medicine, Bule Hora University Institute of Health, Bule Hora University, Bule Hora, Ethiopia; ^10^ Department of Biotechnology and Bioinformatics, JSS Academy of Higher Education and Research, Mysore, Karnataka, India; ^11^ School of Physical Sciences, Amrita Vishwa Vidyapeetham, Mysuru Campus, Mysore, Karnataka, India

**Keywords:** Salacia oblonga, green synthesis, nanoparticles, biomaterials, FT-IR

## Abstract

**Introduction:** The use of plant extracts in the green synthesis of metallic nanoparticles is one of the simplest, most practical, economical, and ecologically friendly methods for avoiding the use of toxic chemicals.

**Method:** Silver nanoparticles (AgNPs) were synthesized, employing a high-efficiency, non- toxic, cost-effective, green, and simple technique that included the use of *Salacia oblonga* root extract (SOR) as a capping agent compared to synthetic nanoparticles. The use of *S. oblonga* can be seen in traditional medicines for treating diabetes, obesity, rheumatism, gonorrhea, asthma, and hyperglycemia. The objectives of the current study were to green synthesize *S. oblonga* root extract silver nanoparticles (SOR-AgNPs), characterize them, and study their antioxidant, antibacterial, and antidiabetic activities.

**Result:** The shape of SOR-AgNPs was spherical, at less than 99.8 nm in size, and exhibited a crystalline peak at XRD. The green synthesized SOR-AgNPs showed significant antioxidant properties like DPPH (80.64 μg/mL), reducing power capacity (81.09 ± SEM μg/mL), nitric oxide (96.58 μg/mL), and hydroxyl (58.38 μg/mL) radical scavenging activities. The MIC of SOR-AgNPs was lower in gram-positive bacteria. The SOR-AgNPs have displayed efficient inhibitory activity against α-amylase, with an EC50 of 58.38 μg/mL. Analysis of capping protein around the SOR-AgNPs showed a molecular weight of 30 kDa.

**Discussion:** These SOR-AgNPs could be used as antibacterial and antidiabetic drugs in the future as it is cheap, non-toxic, and environmentally friendly. Bio-fabricated AgNPs had a significant impact on bacterial strains and could be used as a starting point for future antibacterial drug development.

## 1 Introduction

Scientists from all over the world are focusing on nano-bioscience, due to its potential in many applications, including the ability to fight bacterial resistance. A novel and promising alternative is the use of nanotechnology in the development of potent antibiotics. Silver’s ability to inhibit the growth of bacteria is already well known, but research into nano-sized silver (100 nm) has expanded because of its wide range of applications and exceptional resistance to both Gram-positive and Gram-negative types of bacteria (Salayová et al., 2021). Nanoparticles (NPs) are atomic solid particles that are 100 nm in size and have superior physical properties, based on their size and form (Salayová et al., 2021). Green synthesis of metallic nanoparticles employing plant extracts is one of the easiest, most convenient, cost-effective, and environmentally beneficial approaches for eliminating the use of harmful chemicals. As a result, various environmentally friendly procedures for the fast synthesis of AgNPs employing aqueous extracts of all the parts of the plant have been reported in recent years (Vanlalveni et al., 2021). Reactive oxygen species (ROS) and free radical formation, adherence to microbial cells, penetration inside the cells, or modification of microbial signal transduction pathways are only a few examples of the different processes that could be involved in the mode of action (Salayová et al., 2021). Nanoscience is garnering a lot of interest as a new branch of research that deals with the production of nanoparticles and nanomaterials for use in a variety of sectors such as electrochemistry, catalysis, pharmaceuticals, biomedicine, food technology, sensors, and cosmetics, among others (Bouafia et al., 2021). Many medicinal plants such as *Berberis vulgaris* (Deepak et al., 2020), *Ipomoea carnea Jacq.* (Singh and Navneet, 2021), *Tectona grandis* (Rautela et al., 2019), *Azadirachta indica* (Ahmed et al., 2016), *Salvia spinosa* (Pirtarighat et al., 2018), and *Astragalus tribuloides* Delile (Sharifi-Rad et al., 2020) have been explored for the Phyto-fabrication of AgNPs. Silver must be in its ionized form for it to have any antibacterial properties. Silver is inert and, while it is not ionized, when it comes into contact with moisture, it produces silver ions. When interacting with nucleic acids, Ag + ions prefer to engage with their nucleosides rather than with their phosphate groups, forming complexes with them (Deepak et al., 2020; Salayová et al., 2021).

*Salacia oblonga (SO)*, also known as saptrangi and ponkoranti, is a *Celastraceae* plant native to India, Sri Lanka, China, and other Southeast Asian countries. *SO* has been used as an ayurvedic herb in India for thousands of years. α-glycosidase converts carbohydrates into glucose. The principal use of *SO* is in diabetes research; *S. oblonga* root (SOR) extract binds to the enzymes of the intestine accountable for the breakdown of carbohydrates in the body (Deepak et al., 2020). α -glycosidase enzymes convert carbohydrates to glucose, a sugar that circulates throughout the body. Strong α-glucosidase inhibitors, salacinol and kotalanol, competitively bind to glucosidases in the small intestine (Deepak et al., 2020). If the enzyme attaches to the SO herbal extract instead of the carbohydrate, less glucose enters the bloodstream, resulting in a low blood glucose level. Various phytoconstituents found in *SO* extracts, such as salcinol, kotanol, and mangiferin, have been shown to have anti-diabetic properties (Basu et al., 2013). In this study, the aqueous root extract of *SO* was used to synthesize AgNPs in a completely green manner to supplement the limited literature on *SO* pharmacognostic potential.

## 2 Materials and methods

### 2.1 Collection and preparation of obtained aqueous root extract of *S. oblonga*

The dried roots of *S. oblonga* were collected in Mysore District, Karnataka. Prof. G. R. Shivamurthy, Chairman of Botany, Department of Botany, University of Mysore, Mysuru, authenticated the plant specimen. The collected root material was gently rinsed with distilled water and 10 gm was then weighed out and placed in a Soxhlet extractor for 16 h of consecutive solvent extraction. The obtained aqueous root extract of *S. oblonga* (SOR) was filtered using 0.2M filters (Evans and Trease, 1989). The qualitative phytochemical screening of SOR was performed using standard procedures (Harborne, 1984).

### 2.2 Synthesis of SOR-AgNPs

Using green synthesis methods, AgNPs were successfully synthesized using SOR as a capping and reducing agent and 100 mL of silver nitrate (AgNO₃) as a precursor (Adeleke et al., 2017). To synthesize AgNPs, a volumetric flask was filled with 2 mM 100 mL AgNO₃ and 30 mL of SOR. The AgNPs were synthesized in a magnetic stirrer at 600 rpm at 80°C for 3–4 h. A color change from colorless to brown indicated the synthesis of silver nanoparticles. The UV–Visible spectra of the reaction solution verified the reduction of Ag + ions, confirming the synthesis of silver nanoparticles. Finally, silver nanoparticles were separated from the mixture by centrifugation at 5,000 rpm for 15 min and were washed with methanol, freeze-dried with a Lyophilizer, and kept at 4°C for further use (Senguttuvan et al., 2014).

### 2.3 Characterization of SOR-AgNPs

Understanding and controlling NP manufacturing and application requires the characterization of silver nanoparticles (Abou El-Nour et al., 2012). The synthesis of silver nanoparticles was affirmed by sampling the reaction mix at frequent intervals and scanning the absorption maximum with UV-Vis spectra between 350 and 700 nm using a HITACHI U-2900 Double beam spectrometer. The functional biomolecules in *S. oblonga* that were responsible for the metal reduction and nanoparticle stabilization were classified using FTIR RX1-Perkin Elmer in the 4,000–400 cm^−1^ wavelength range. XRD was used for the determination of the crystalline nature of the AgNPs, and patterns of the AgNPs were estimated using the Smart Lab 3Kw. A DLS particle size analyzer was used to estimate the size distribution and average size of AgNPs. Energy dispersive X-RAY analysis was utilized to estimate the elemental makeup. SEM was used for examining the structure of AgNPs (HITACHI S-3400N).

### 2.4 Assessment of antioxidant activity of SOR-AgNPs

#### 2.4.1 2, 2-Diphenyl-1-Picryl-hydrazyl (DPPH) free radical scavenging assay

DPPH free radical scavenging ability of the silver nanoparticles was investigated (Flieger et al., 2021). SOR-AgNPs were combined with 1 mL of DPPH (0.1 mM) dissolved in methanol at varying concentrations, followed by vortexing and mL incubating for 30 min in a dark environment. At 517 nm, the absorbance of stable DPPH was measured. The same technique was utilized to prepare the DPPH (which contained no sample). As a reference standard, standard butylated hydroxytoluene (BHT) was used. The IC₅₀ value was calculated by plotting the percentage inhibition vs. concentration. Using the following formula, the inhibition percentage was obtained and represented as RSA% (Percentage of Radical Scavenging Activity).

$$RSA\%=\frac{\left({OD}_{control}-{OD}_{sample}\right)}{{OD}_{control}}\times 100$$

#### 2.4.2 Nitric oxide radical scavenging assay

Nitric Oxide radical scavenging assay was estimated by preparing (Senguttuvan et al., 2014) different concentrations of SOR-AgNPs and making them up to 1 mL by adding distilled water, followed by the addition of 1 mL 10 mM sodium nitroprusside and 1 mL Griess reagent. The contents were thoroughly vortexed before being incubated at room temperature for 1 h. The appearance of a pink chromophore in diffused light was observed and absorbance was compared to that of blank solutions at 540 nm. The activity was calculated using the previously mentioned formula.

#### 2.4.3 Reducing power capacity

Reducing power was determined by preparing (Keshari et al., 2021) different concentrations of SOR-AgNPs (20, 40, 60, 80, and 100 μg/mL). 2.5 mL of 0.01M sodium phosphate buffer, pH 7.5, was supplemented in these extracts, followed by 2.5 mL of 1% potassium ferricyanide (K_3_Fe(CN)_6_) solution. Before being incubated at 50°C for 20 min, the contents were vortexed vigorously. After the incubation period, each tube received 2.5 mL of 10% TCA and was centrifuged for 10 min at 3,000 rpm. Each tube received 5 mL of the supernatant, 5 mL of deionized water, and 1 mL of 1% FeCl_3_ and was incubated at 35°C for 10 min. The extract’s reducing power was proportional to the sample’s concentration. The reaction mixture’s increasing absorbance indicated a power reduction. As a reference standard, butylated hydroxyl toluene (BHT) was used. Absorbance was recorded at 700 nm and the percentage of inhibition was determined using the previously mentioned equation.

#### 2.4.4 Hydroxyl radical scavenging assay (HRSA)

HRSA was determined with minor modifications (Chinnasamy et al., 2021) by adding different concentrations of SOR-AgNPs with 1 mL of iron ethylenediamine tetraacetic acid (EDTA) solution (0.26%EDTA and 0.13% ferrous ammonium sulphatemL), 1 mL of DMSO (0.85%v/v I 0.1 M phosphate buffer pH 7.4) and 0.5 mL of EDTA solution (0.018%). This reaction was started by the addition of 0.5 mL of ascorbic acid (0.22%) and incubating this solution in the water bath for 15 min at 80–90°C. The reaction was halted by adding 1 mL of ice-cold TCA (17.5 percent w/v) after it had been incubated. 3 mL Nash reagent was added and allowed to sit for 15 min at room temperature. In comparison to the reagent blank, the identification of the color developed was recorded spectrophotometrically at 412 nm. The percentage of HRSA activity was calculated using the formula previously mentioned.

### 2.5 Antibacterial assay

The antibacterial activity of the SOR-AgNPs was determined by using the agar disc diffusion method (Saratale et al., 2017). The antimicrobial property of the SOR-AgNPs was determined against *Bacillus subtilis, Escherichia coli, Bacillus cereus, Salmonella typhi, and Staphylococcus aureus*. Inoculum containing around 150 × 10^4^ CFU/mL was swabbed uniformly onto the nutrient agar (NA) coated plates and left to incubate in a sterilized atmosphere. A 6 mm sterilized disc was loaded with various dilutions of SOR-AgNPs (1, 5, 10, and 15 μg/mL) solutions, while another disc emersed in 1 μg/mL of ampicillin was considered a positive control. To determine the zone of inhibition, the Petri dishes were incubated at 36°C for 2 days. The average was derived by repeating the experiments three times.

### 2.6 *In vitro* anti-diabetic potential

The mixture of α -amylase comprised of 0.5 mL of sodium phosphate buffer (0.02M, pH 6.9) with α-amylase (1U/mL) and differing concentrations of SOR-AgNPs (20–100 μg/mL) that had been pre-incubated at 37°C for 20 min. After incubation, 2.5 mL of 1% starch in phosphate buffer was supplemented and incubated at 37°C for 15 min. This reaction was halted by adding 1 mL of dinitro salicylic acid reagent and heated for 10 min then cooled, and the absorbance was determined at 540 nm (Chowdhury et al., 2014). The activity was calculated with a 0.01 increase in absorbance per mL/min.

### 2.7 Investigation of capping protein around the SOR-AgNPs

The method developed by Chowdhury et al. (Macovei et al., 2021) was used to cap the protein, with minor modifications. The protein(s) attached to the surface of SOR-AgNPs were isolated by washing them with sterile distilled water and boiling them for 10 min with 1% SDS (sodium dodecyl sulfate). Electrophoresis was carried out in 12% SDS-PAGE at a constant voltage of 70 V for 120 min. Coomassie Brilliant blue dye was used to stain the gel for observation.

### 2.8 Statistical analysis

The outcomes were calculated as mean ± SD of three trials (*p* < 0.01). The DPPH assay’s IC_50_ values were calculated and statistically analyzed.

## 3 Results and discussion

### 3.1 Phytochemical screening of *S. oblonga*

The phytochemical screening was done qualitatively using SOR, which contained lead phytoconstituents, such as alkaloids, flavonoids, saponins, phenols, tannin, phytosterols, and vitamin C, proteins, amino acids, and terpenoids. These phytoconstituents are important to lead molecules involved in nanoparticle production. When the findings were compared to earlier studies, it was discovered that Phyto-functionalized silver nanoparticles contained more phytoconstituents from conifer bark extract (Melkamu and Bitew, 2021).

### 3.2 SOR-AgNPs biosynthesis and UV-Visible spectroscopy analysis

UV-Visible spectroscopy was used in this investigation to monitor the process of silver ion bioreduction to AgNPs. It was used to monitor the creation and optimization of AgNPs by monitoring absorbance in the 300–700 nm scanning range. The development of a distinctive Surface Plasmon Resonance (SPR) peak of 448 nm confirms the presence of AgNPs (Sharifi-Rad et al., 2021). Owing to the excitation of electrons and variations in electronic energy levels, the synthesized SOR-AgNPs will have a dark-brown appearance in an aqueous solution, conforming to the formation of the nanoparticles (Jyoti et al., 2016).

### 3.3 Analysis using a scanning electron microscope (SEM)

**FIGURE 1:**
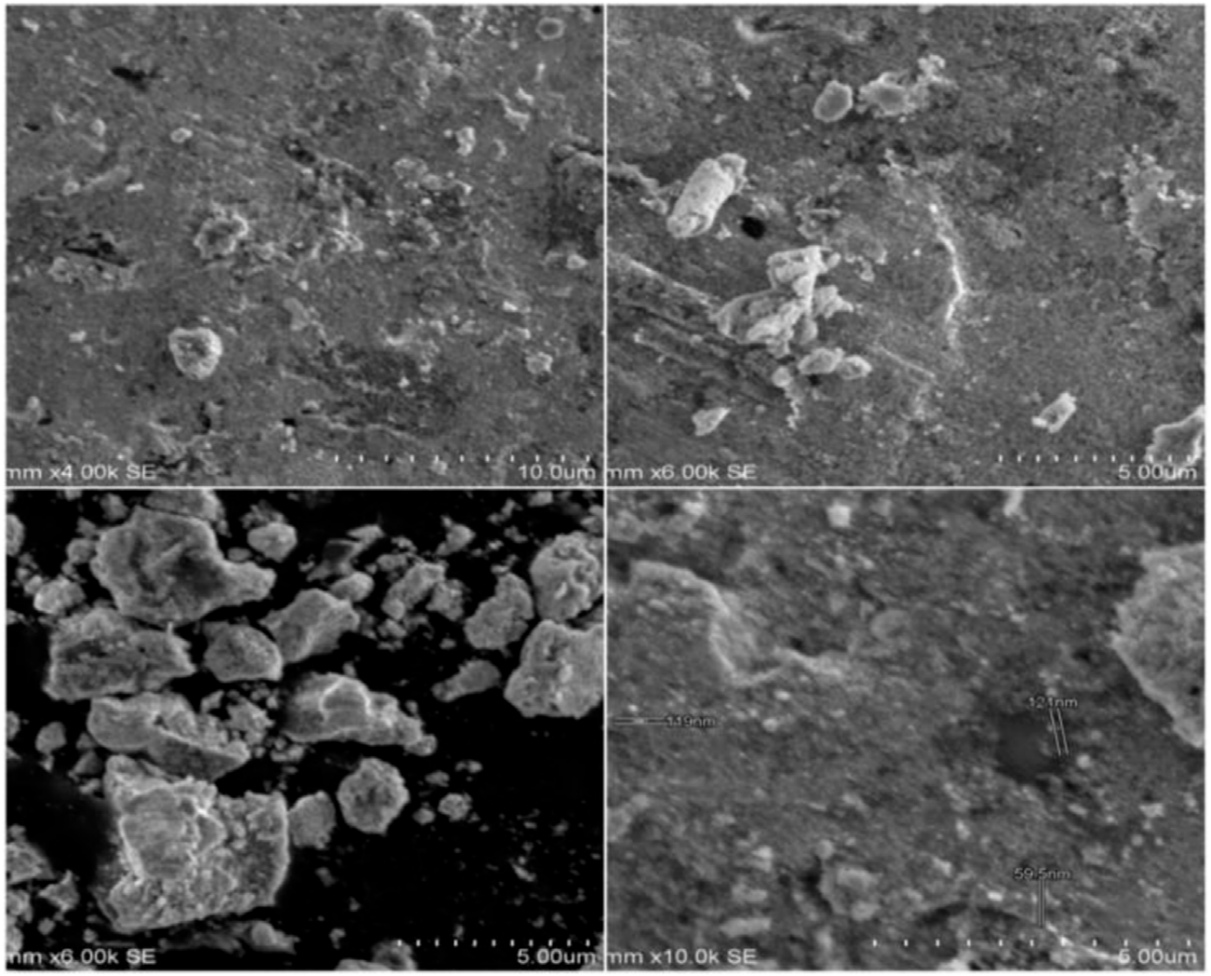
SEM images of as-prepared SOR-AgNPs in different magnifications.

SEM analysis of the morphology (viz size and shape) of SOR-AgNPs showed multiform nanoparticles having a spherical, crystal, and rode-like appearance. As depicted in Figure 1, the shapes had an average size range of between 59 and 78 nm. Even within the aggregates, the nanoparticles were not in direct contact, indicating that the nanoparticles were stabilized by a capping agent. According to the previous report, SEM demonstrated the spherical nature of silver metal particles generated in *Urtica dioica Linn* leaf extract (Blois, 1958).

### 3.4 Capping protein analysis around the AgNPs

**FIGURE 2:**
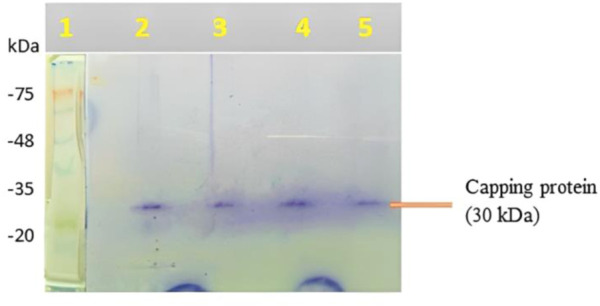
SDS-PAGE of capping protein upon treatment with as-prepared SOR-AgNPs.

1st lane, molecular size marker; 2nd lane, NP (10 g/mL); 3rd lane, NP (20 g/mL); 4th lane, NP (30 g/mL); and 5th lane, NP (40 g/mL) were demonstrated in the SDS-PAGE study of capping protein surrounding AgNPs generated from the aqueous root extract of *S. oblonga* (Figure 2). In the aqueous root extract of *S. oblonga*, there is a strong protein band (30 kDa) that is primarily involved in protein capping and offers stability for the bio-reduction of AgNO_3_ to AgNPs.

### 3.5 Time kinetics of synthesized AgNPs at various stirring time intervals

**FIGURE 3:**
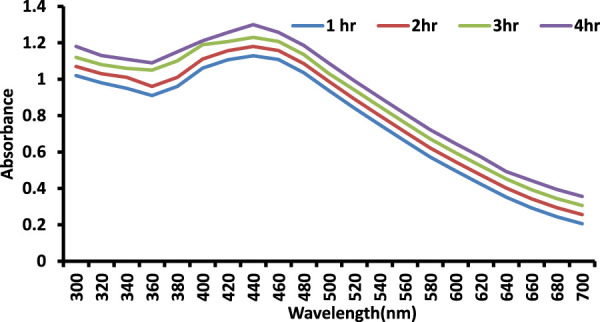
Time kinetics study of as-prepared SOR-AgNPs.

**FIGURE 4:**
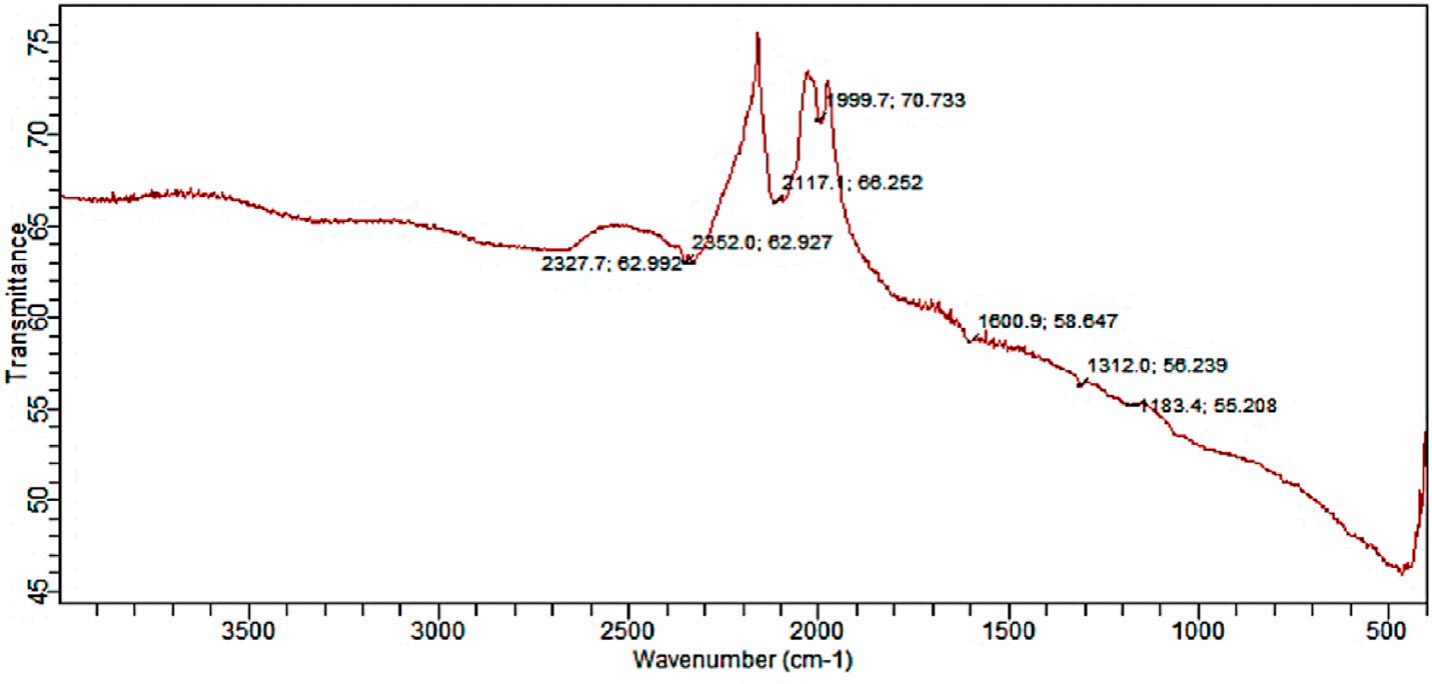
FT-IR spectrum indicating important stretching frequencies in SOR-AgNPs.

The influence of stirring time on the bioproduction of AgNPs is depicted in the diagram (Figure 4). With increasing stirring time, the color intensity and monodispersity increased. The emergence of an SPR peak in the 400–450 nm wavelength range correlates to AgNPs, which absorb heavily at the 426 nm wavelength (Figure 3). When compared to previous reports, the stability of AgNPs rises after 72 h of stirring time.

### 3.6 FT-IR spectra of biosynthesized SOR-AgNPs

The existence of protein stabilizing molecules was shown by FT-IR analysis, which revealed a peak range of 500–4,000 cm^−1^, all of which corresponded to distinct functional groups. The results of the FTIR study demonstrate that various functional groups have sharp absorption peaks at 2327, 2352, 2117, 1999, 1600, 1312, and 1183 cm^−1^ (Figure 4). The different absorption peaks, such as those at 1000–1300 cm^−1^, which could be aldehyde or ketone; 1999 cm^−1^, which could be aromatics; and 2300 cm^−1^, which could be the amide bond of proteins, are caused by carbonyl stretching in proteins and by the interaction of SOR-AgNPs through green synthesis, and the secondary structure was unaffected during the reaction with silver ions or post binding with AgNPs. The carbonyl group has a strong silver binding ability, indicating the formation of SOR-AgNP covering layers and acting as a capping mediator to prevent agglomeration and give the medium strength, according to this study. These findings support the existence of proteins that act as reducing and stabilizing agents (Anandalakshmi et al., 2016).

### 3.7 Energy dispersive X-ray (EDX) spectra analysis

**FIGURE 5:**
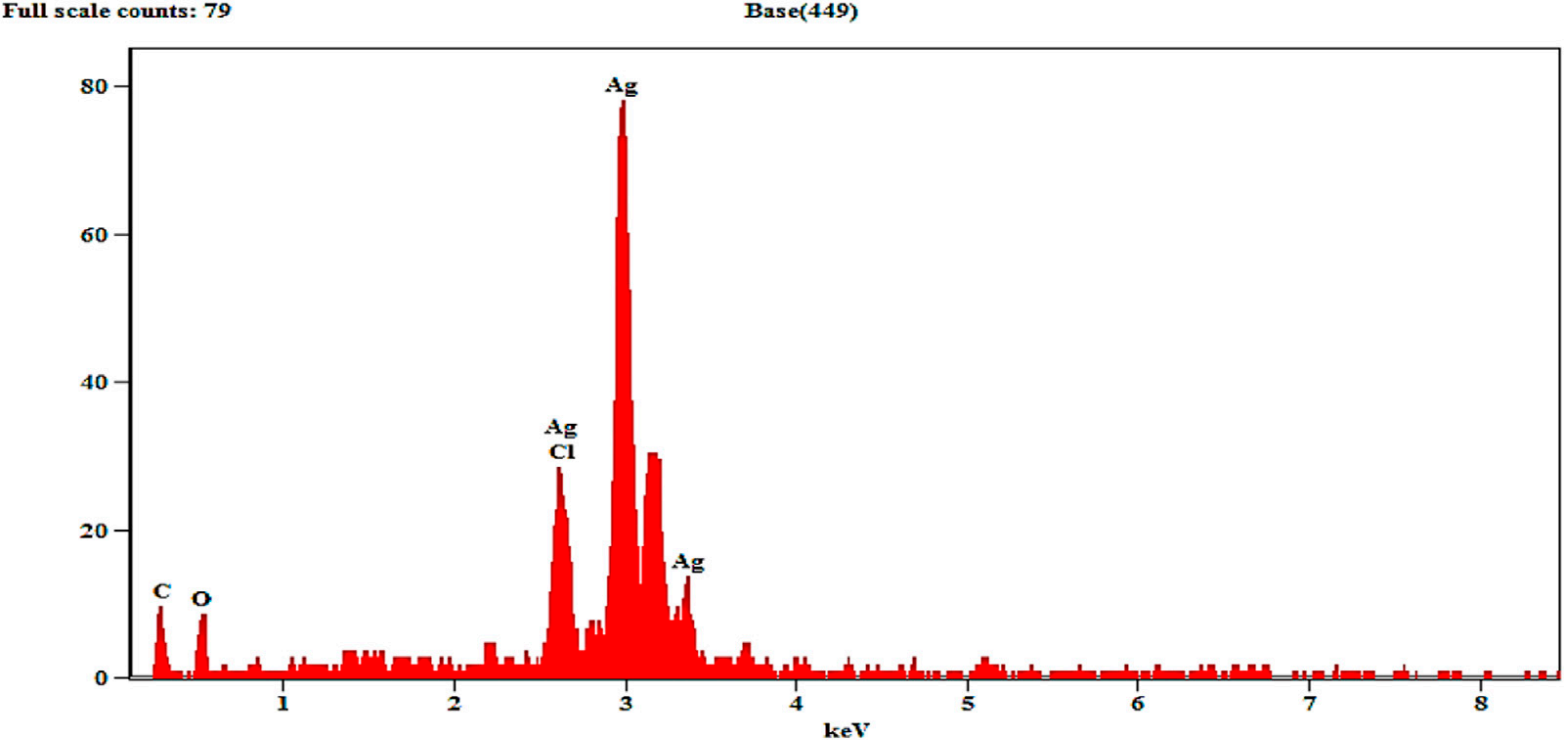
Energy dispersive X-ray spectrum of as-prepared SOR-AgNPs.

EDX determines the quantitative and qualitative properties of the elements involved in AgNP production. The peak in the silver region at 3 KeV (80 in mass) is usually for the absorption of metal silver nanocrystalline owing to SPR, as can be seen in Figure 5. Additional signals from atoms N, O, C, and Cl were documented. Similar results were confirmed in the elemental profile of synthesized NPs using banana peel extract, showing greater counts at 3 keV because of silver and affirming the development of AgNPs (Ibrahim, 2015).

### 3.8 X-ray diffraction (XRD) spectrum measurement

**FIGURE 6:**
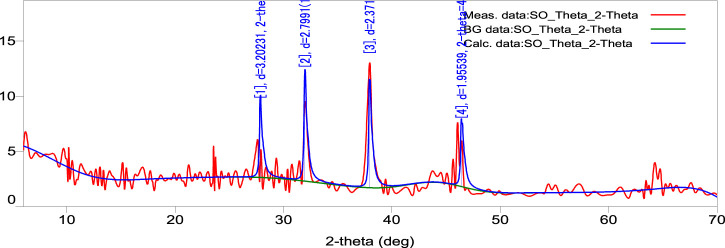
XRD pattern of as-prepared SOR-AgNPs.

The XRD pattern of the biosynthesized AgNPs is depicted in Figure 6. The (4.202), (2.799), (2.371), and (1.955) reflections of metallic silver were revealed by the four diffraction peaks at 28.00˚, 32.23˚, 38.25˚, and 46.48˚. A strong and crisp diffraction peak positioned at 32.23˚ was observed, which can be linked to the (2.799). The strong peaks clearly show that the SOR-AgNPs produced have a face-centered cubic (FCC) shape. The bioorganic phase crystallizes on the silver nanoparticles’ surfaces, according to other peaks. The results were compared to the synthesis of AgNPs from the seed extract of *Tectona grandis* and an XRD pattern of AgNPs, both of which corroborated the crystalline nature of AgNPs. The four different diffraction peaks at four values of 38.05, 44.23, 64.41, and 76.66 may be indexed to the face-centered cubical structure of silver (111), (200), (220), and (311) reflection planes (Rautela et al., 2019).

### 3.9 Dynamic light scattering (DLS) measurement

**FIGURE 7:**
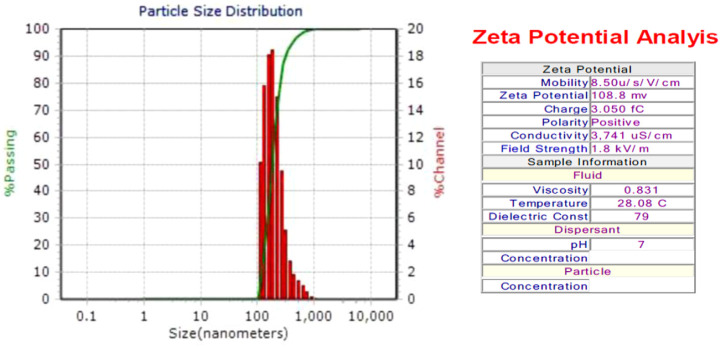
DLS measurement of as-prepared SOR-AgNPs.

The diameter of SOR-AgNPs is 181.5 nm, according to the size distribution histogram of DLS (Figure 7). The sample’s Zeta potential analysis revealed that the positive polarity of the particles favors drug targeting (Clogston and Patri, 2011).

### 3.10 Antioxidant potential of SOR-AgNPs

**FIGURE 8:**
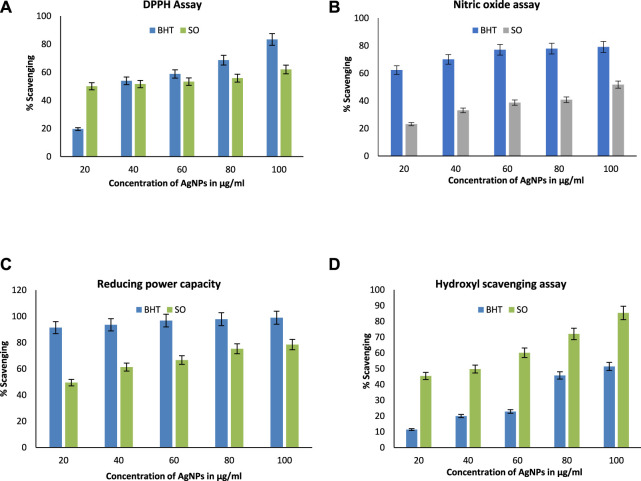
Antioxidant activity **(A)** DPPH radical scavenging assay; **(B)** nitric oxide radical scavenging assay; **(C)** reducing power assay, and **(D)** hydroxyl radical scavenging assay.

Antioxidants are compounds that fight free radicals. Antioxidants are natural or manmade chemicals that can prevent or postpone oxidant-induced (ROS, RNS, free radicals, and other unstable molecules) cell damage (Azeez et al., 2017). ROS is involved in the etiology of several degenerative disorders, along with cardiovascular disease and cancer. In this study, the radical-scavenging effects were investigated using DPPH with a distinctive absorption at 517 nm. When compared to the traditional BHT scavenging assay, the DPPH scavenging assay showed efficient SOR-AgNPs inhibition (butylated hydroxytoluene). The antioxidant activity (DPPH method) reveals that SOR-AgNPs had an IC_50_ value of (80.64 g/mL), whereas conventional BHT is 60 g/mL, as shown in (Figure 8A). According to a previous study, green synthesis of silver nanoparticles using *Prosopis farcta* fruit extract exhibited potential antioxidant activity in comparison with ascorbic acid, which was considered the standard (Salari et al., 2019). For Nitric oxide radical scavenging activity (Figure 8B), the SOR-AgNP activity was significant (96.58 g/mL) when compared to standard BHT (Sudha et al., 2017). SOR-AgNPs extract has significant reducing power activity, and the increasing SOR-AgNP concentration continuously increased reducing power activity. SOR-AgNPs (81.09 g/mL) and normal BHT (54.71 g/mL) exhibit nearly identical reducing power activity, as shown in (Figure 8C). Similarly, in the hydroxyl scavenging assay, scavenging potential increased with an increase in the concentration of BHT and SOR-AgNPs (Figure 8D). The presence of phytoconstituents in the leaf extract resulted in the activity of reducing power (Bhakya et al., 2016).

### 3.11 α-Amylase inhibition assay

**FIGURE 9:**
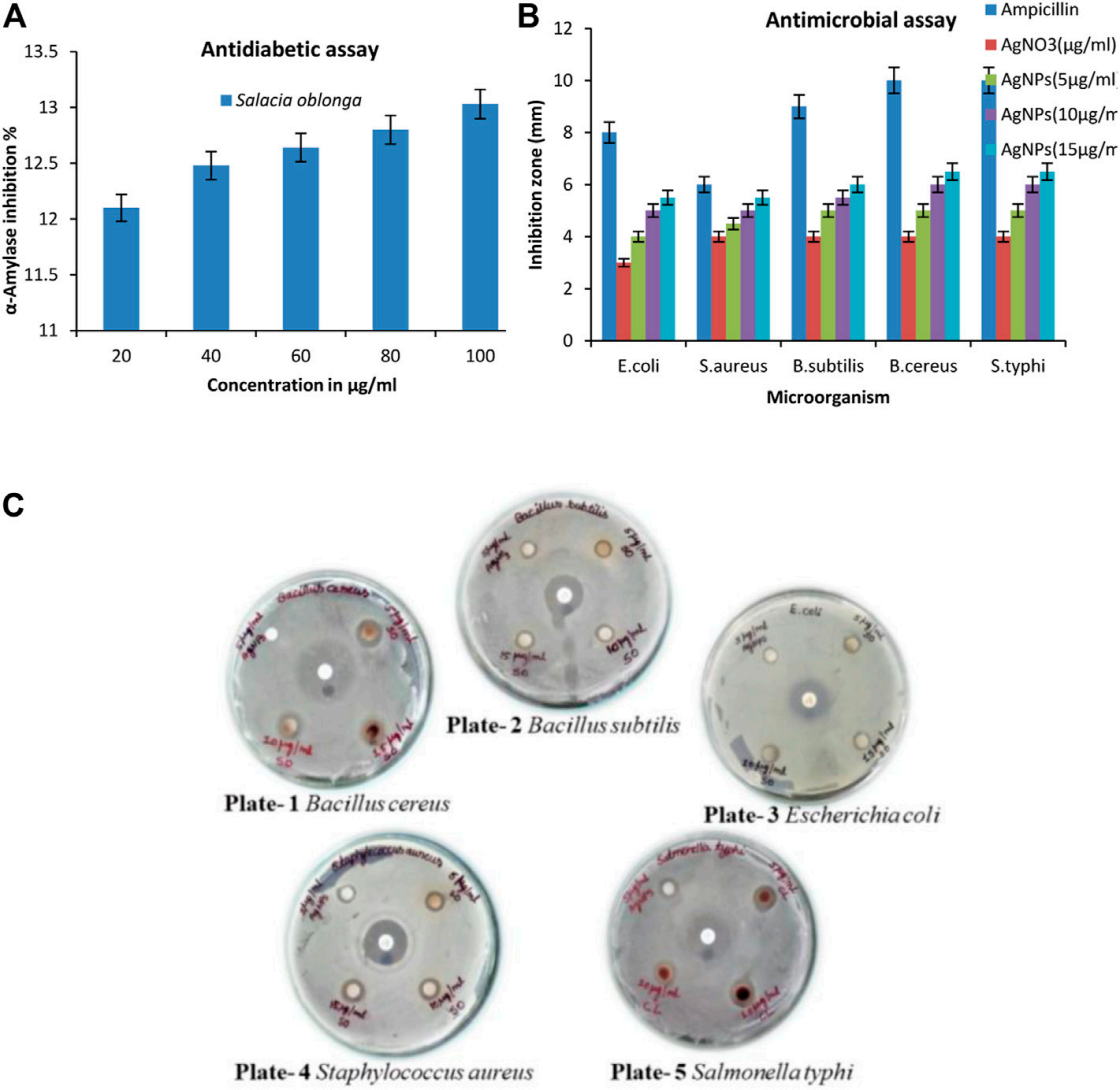
**(A)** *In vitro* antidiabetic activity (α-amylase assay) by SOR-AgNPs. 20–100 μg/mL of SOR-AgNPs were pre-incubated with α -amylase (1U/mL) at 37°C for 20 min. After incubation, 1% starch solution was added to the tubes and incubated at 37°C for 15 min. Absorbance was measured at 540 nm and inhibition activity was calculated and represented as Mean ± SD (*n* = 3); **(B)** Graph of antimicrobial assay of the SOR-AgNPs. Inoculums (150 × 10^4^ CFU/mL) were swabbed uniformly onto nutrient agar plates, and a 6 mm sterile disc was loaded with various concentrations of SOR-AgNPs. The Petri dishes were then incubated at 26°C for 48 h to calculate the inhibition zone and values signified as Mean ± SD (*n* = 3); **(C)** Evaluation of the antibacterial potential SOR-AgNPs. Plate -1: *Bacillus cereus* [a) Standard antibiotic, b) 5 μg/mL AgNPs, c) 5 μg/mL SOR, d) 15 μg/mL SOR e) 10 μg/mL SOR]. Plate -2: *Bacillus subtilis* [a) Standard antibiotic, b) 5 μg/mL AgNPs, c) 5 μg/mL SOR, d) 10 μg/mL SOR e) 15 μg/mL SOR]. Plate -3: *Escherichia coli* [a) Standard antibiotic, b) 5 μg/mL AgNPs, c) 5 μg/mL SOR, d) 15 μg/mL SOR e) 10 μg/mL SOR]. Plate -4: *Staphylococcus aureus* [a) Standard antibiotic, b) 5 μg/mL AgNPs, c) 5 μg/mL SOR, d) 10 μg/mL SOR e) 15 μg/mL SOR]. Plate -5: *Salmonella typhi* [a) Standard antibiotic, b) 5 μg/mL AgNPs, c) 5 μg/mL CL, d) 15 μg/mL CL e) 10 μg/mL CL].

The carbohydrate-hydrolyzing enzyme α-amylase was substantially inhibited by SOR-AgNPs. The level of enzymatic activity was dramatically reduced as the concentration of SOR-AgNPs increased (Figure 9A). The EC_50_ values for SOR-AgNP inhibition of α-amylase were 58.38 g/mL, and previous research has described the green synthesis of AgNO_3_ utilizing the leaf extract of *Calophyllum tomentosum* with antidiabetic action (Govindappa et al., 2018).

### 3.12 Assessment of the antibacterial property

The antibacterial efficacy of SOR-AgNPs is powerful against bacterial species. AgNPs showed antibacterial activity in different degrees, as indicated by the inhibition zone diameter, but SOR-AgNPs had much stronger antibacterial activity than AgNPs (Figures 9B, C). When compared to previous research, the results show that aqueous callus extracts of *Fagonia indica* silver nanoparticles have a stronger antibacterial effect (Adil et al., 2019).

## 4 Conclusion

In summary, the present work defines the formation of AgNPs using an aqueous root extract of *S. oblonga*. The root extract of *S. oblonga* comprised of phytocompounds accountable for the capping and bio-reduction of AgNO_3_ into AgNPs. Stability to these nanoparticles is provided by the capping agent. The synthesized SOR-AgNPs have shown hydroxy radical, nitric oxide radical, antioxidant, and reducing power activities owing to the incidence of functional groups on the surfaces of AgNPs. Because of their tiny size and the existence of capping agents, green synthesized SOR-AgNPs show significant antimicrobial properties against specific human pathogenic microbes and more powerful *in vitro* antidiabetic action. These SOR-AgNPs could be used as antibacterial and antidiabetic drugs in the future as it is cheap, non-toxic, and environmentally friendly. As a result, this technology can be utilized to make large-scale nanoparticles, which can be used in a variety of medical and technical applications.

## Data Availability

The original contributions presented in the study are included in the article/Supplementary Material, further inquiries can be directed to the corresponding authors.
